# Comparative transcriptome analysis reveals key epigenetic targets in SARS-CoV-2 infection

**DOI:** 10.1038/s41540-021-00181-x

**Published:** 2021-05-24

**Authors:** Marisol Salgado-Albarrán, Erick I. Navarro-Delgado, Aylin Del Moral-Morales, Nicolas Alcaraz, Jan Baumbach, Rodrigo González-Barrios, Ernesto Soto-Reyes

**Affiliations:** 1 Departamento de Ciencias Naturales, Universidad Autónoma Metropolitana-Cuajimalpa (UAM-C), Mexico City, Mexico; 2 Chair of Experimental Bioinformatics, TUM School of Life Sciences Weihenstephan, Technical University of Munich, Munich, Germany; 3 Unidad de Investigación Biomédica en Cáncer, Instituto Nacional de Cancerología, Mexico City, Mexico; 4 The Bioinformatics Centre, Department of Biology, University of Copenhagen, Copenhagen, Denmark; 5 Chair of Computational Systems Biology, University of Hamburg, Hamburg, Germany; 6 Computational BioMedicine Lab, Institute of Mathematics and Computer Science, University of Southern Denmark, Odense, Denmark

**Keywords:** Systems analysis, Regulatory networks

## Abstract

COVID-19 is an infection caused by SARS-CoV-2 (Severe Acute Respiratory Syndrome coronavirus 2), which has caused a global outbreak. Current research efforts are focused on the understanding of the molecular mechanisms involved in SARS-CoV-2 infection in order to propose drug-based therapeutic options. Transcriptional changes due to epigenetic regulation are key host cell responses to viral infection and have been studied in SARS-CoV and MERS-CoV; however, such changes are not fully described for SARS-CoV-2. In this study, we analyzed multiple transcriptomes obtained from cell lines infected with MERS-CoV, SARS-CoV, and SARS-CoV-2, and from COVID-19 patient-derived samples. Using integrative analyses of gene co-expression networks and de-novo pathway enrichment, we characterize different gene modules and protein pathways enriched with Transcription Factors or Epifactors relevant for SARS-CoV-2 infection. We identified EP300, MOV10, RELA, and TRIM25 as top candidates, and more than 60 additional proteins involved in the epigenetic response during viral infection that has therapeutic potential. Our results show that targeting the epigenetic machinery could be a feasible alternative to treat COVID-19.

## Introduction

The coronavirus family (CoV) are non-segmented, positive-sense, and enveloped RNA viruses that have been identified as the cause of multiple enteric and respiratory diseases in both animals and humans^1^. Three major CoV strains of this family have caused recent human pandemics: the Middle East respiratory syndrome coronavirus (MERS-CoV) in 2002–2003^2^, severe acute respiratory syndrome coronavirus 1 (SARS-CoV) in 2012, and SARS-CoV-2 in 2020^3^. The most recent one was identified in Wuhan, China by the end of 2019 and is the etiological origin of atypical pneumonia known as Coronavirus Disease 2019 (COVID-19), which has caused a global outbreak and is one of the top sixth public health emergencies of international concern^4^ with 98,089,877 confirmed cases and 2,100,404 deaths as of January 2021, leading to the biggest CoV pandemic in modern times^5^.

By being intracellular pathogens, viruses’ infection strategy requires the continuous subordination and exploitation of cellular transcriptional machinery and metabolism in order to ensure its own expansion. To do so, the host genome expression must be used and, to be successful, this will depend on chromatin dynamics and transcription regulation, which are principally ruled by epigenetic mechanisms, such as DNA methylation, histone post-translational modifications (HPTM), and transcription factors (TFs)^6^. During a viral infection, it has been reported that epigenetic and transcriptional changes occur for both sides: the infected cell promotes an antiviral environmental response, leading to the induction of pathways to survive, while the virus switches off the expression of critical anti-viral host cell genes^7,8^.

Several studies have reported the importance of epigenetic modifications in viral infections. In the influenza virus, specific gene promoter DNA methylation^9^, decreased H3K4me3 (a hallmark of active chromatin)^10^, histone acetylation in H3 and H4 histones, and increased levels of H4K20me2 and unmodified H3K36 and H4K79 have been reported^11^. Interestingly, these HPTMs do not always trigger the same mechanisms and lead to similar phenotypes; for example, depletion of H3K79me2, an epigenetic mark that is usually increased upon viral infections due to an upregulation of DOT1L, results in impaired viral growth in human cytomegalovirus infection^12^, while enhancing the replication in influenza virus^11^. However, these mechanisms usually lead to host transcriptional inactivation, which contributes to the altered cellular transcription produced by viral infections.

Regarding CoVs, few experimental studies have been conducted to unravel the epigenetic proteins and marks involved in their infection and pathogenesis in MERS-CoV and SARS-CoV, being especially scarce in SARS-CoV-2 due to its recent appearance. For MERS-CoV and SARS-CoV, different outcomes have been reported, such as the mechanisms used to control the interferon-stimulated genes, which involve H3K27 methylation in MERS-CoV but not in SARS-CoV^13^, and the ones used to down-regulate antigen-presenting molecules, which involves DNA methylation in MERS-CoV and not in SARS-CoV^9^. These studies show that epigenetic mechanisms are highly important in the host gene expression control carried out by the virus and that, despite the phylogenetic closeness, these mechanisms can be very different between strains, highlighting the need to understand the epigenetic processes that play a role in SARS-CoV-2 infection.

Integrative computational methods are promising approaches used to generate research hypotheses, generate consensus regulatory networks and describe deregulated processes in SARS-CoV-2 infection^14,15^. Nevertheless, they have overlooked key epigenetic and TFs that underlie the infected phenotype. Since drugs that target the epigenetic landscape of diseased cells have shown great potential and have proved to be game-changing as complementary treatments of complex diseases, such as cancer^16^, the identification of these key epigenetic proteins and TFs become highly important in our current context, where popular regimen candidates for treating COVID-19, such as Remdesivir, Hydroxychloroquine, Lopinavir, and Interferon have shown to have little or no effect on reducing mortality of hospitalized COVID-19 individuals^17^.

In this work, we gathered publicly available RNA-seq data from SARS-CoV-2, SARS-CoV, and MERS-CoV infected cell lines and patient samples and performed differential expression analyses together with a weighted gene co-expression network analysis to identify unique and shared central epigenetic players in SARS-CoV-2, SARS-CoV, and MERS-CoV. Candidate genes were further prioritized by integrating differentially expressed genes (DEGs), enrichment tests, gene-coexpression network, and viral–host protein–protein interaction network analysis to propose potential key epigenetic proteins involved in SARS-CoV-2 infection. Finally, we identified currently approved drugs that target key epigenetic drivers of SARS-CoV-2 infection, and thus they are potential new therapeutic approaches for COVID-19.

## Results

### SARS-CoV-2, SARS-CoV, and MERS-CoV induce different transcriptional and epigenetic responses during infection in pulmonary cell lines

In order to identify the genes that change their expression in pulmonary cell lines (Calu-3, MRC-5, A549, and NHBE) due to infection of Coronaviruses such as MERS-CoV, SARS-CoV, or SARS-CoV-2, differential expression analysis was performed in RNA-seq data (Supplementary Table 1).

**Fig. 1:**
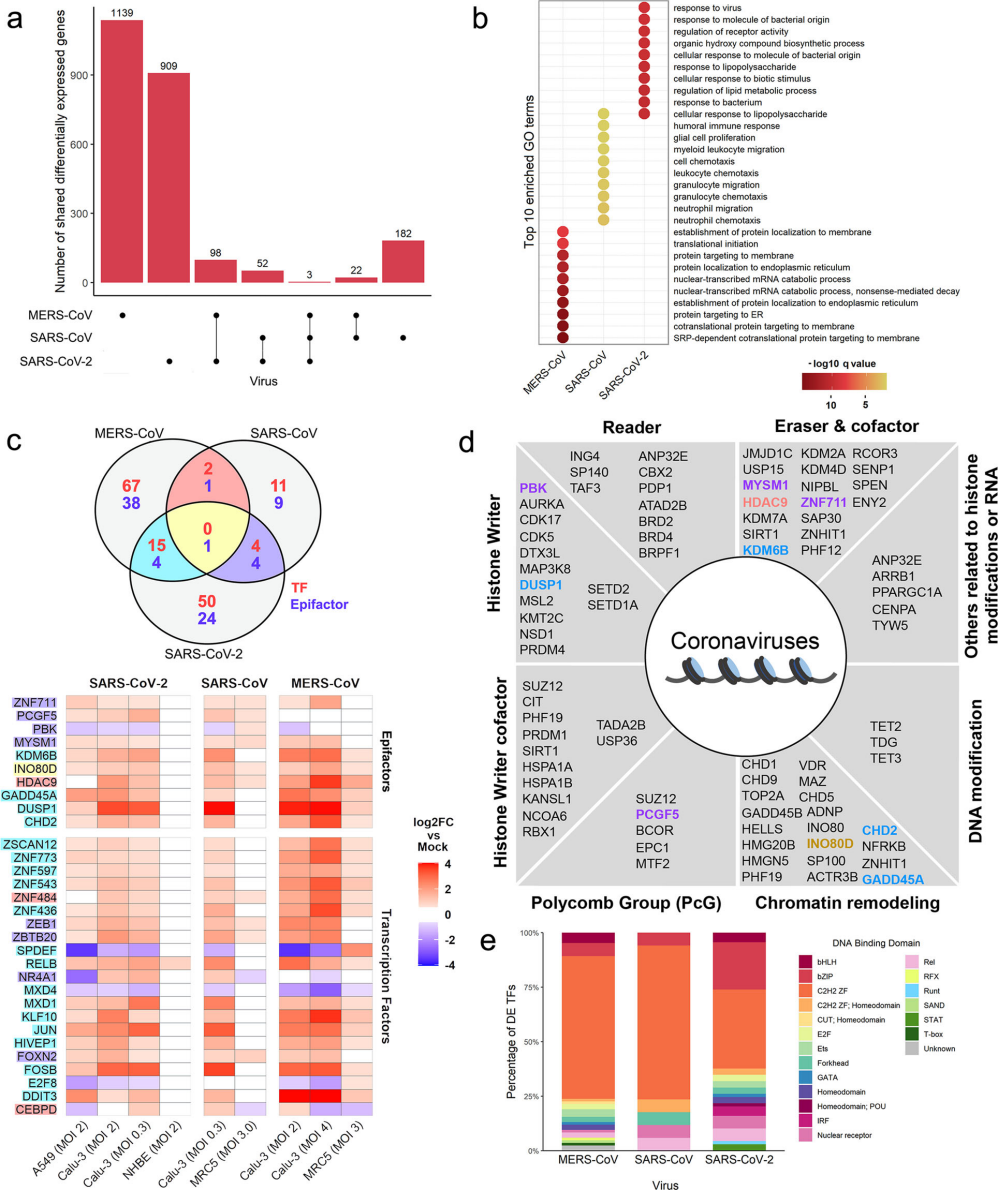
Differential expression analysis of coronavirus-infected cell lines. **a** Intersection size of the DEGs common to each viral infection represented as single dots (virus-associated gene sets) and the size of their intersections with the other sets (multiple vertical dots). **b** Top ten simplified enriched Gene Ontology terms of biological process in the virus-associated gene sets ordered by *q*-value. **c** Shared differentially expressed epigenes between virus-associated gene sets; text color corresponds to the gene classification as either TF (red) or epifactor (blue) (upper panel). Log2 fold change of shared differentially expressed epifactors in each cell line are also shown as a heatmap (lower panel); blank color represents non-significant differential expression, text highlight corresponds to the intersections shown in the Venn diagram. **d** Functional classification of the identified epifactors; text color corresponds to the intersection color of subsection (**c**). **e** Characterization of the DNA-binding domain (DBDs) of human transcription factors (TFs) altered by the viral infection of coronaviruses.

As a first approach, we evaluated the overlapping DEGs identified for each virus regardless of the cell type and in common among viruses (Supplementary Table 2). For MERS-CoV and SARS-CoV, the overlap among all cell conditions was considered. For SARS-CoV-2, the overlap among 3 out of the 4 cell conditions was used, since the NHBE cell line showed a small number of DEGs most likely because these cells are derived from normal bronchial epithelial cells^18,19^ (Supplementary Fig. 1a). We observed that the majority of the virus-associated genes are unique for each virus and a small proportion is shared among them. Specifically, only three genes were differentially expressed during infection in cell lines regardless of the virus evaluated (Fig. 1a). Furthermore, GO enrichment analysis (Fig. 1b) shows that the top ten enriched GO terms are different for each virus, except “cellular response to lipopolysaccharide”, shared between SARS-CoV-2 and SARS-CoV; however, the three viruses share terms related to immune response processes (Supplementary Fig. 1b). The latter shows that, despite their phylogenetic relationship, the main changes in gene expression driven by MERS-CoV, SARS-CoV-2, and SARS-CoV infection are divergent at both levels: at the DEGs and the cellular processes, suggesting that each virus uses specific molecular strategies during infection.

Subsequently, we inspected the DEGs with epigenetic or transcriptional regulatory function present among viruses, hereinafter referred to as epigenes. A comparative analysis of the DEGs among the three viruses revealed that only *INO80D*, a regulatory component of the chromatin remodeling INO80 complex, is shared among them. MERS-CoV and SARS-CoV only share the histone deacetylase *HDAC9*; while MERS-CoV and SARS-CoV-2 share *DUSP1*, *KDM6B*, *CHD2*, and *GADD45A*. Between SARS-CoV and SARS-CoV-2, we found *PBK MYSM1*, *ZNF711*, and *PCGF5* (Fig. 1c, Supplementary Fig. 1c). In addition, given that TFs are also key elements in gene remodeling and regulation, we evaluated the ones differentially expressed across viruses and none of them was affected in all conditions. However, MERS-CoV and SARS-CoV share *ZNF484* and *CEBPD*; SARS-CoV and SARS-CoV-2 share *ZEB1*, *ZEBTB20*, *NR4A1*, and *FOXN2*, and between MERS-CoV and SARS-CoV-2 15 shared TFs were found, including *RELB*, *JUN*, *FOSB*, *E2F8*, among others (Fig. 1c, Supplementary Fig. 1d).

Furthermore, the analysis showed that the differentially expressed epifactors belong to a wide range of functional categories, such as histone writers, histone readers, histone erases, Polycomb group proteins, chromatin remodeling, DNA modifications, among others (Fig. 1d). In addition, regarding differentially expressed TFs, cell lines infected with SARS-CoV-2 show differential expression of TFs of the STAT (mediators of the cellular response to cytokine) and IRF (interferon-regulatory factor) family, which are not differentially expressed in MERS-CoV and SARS-CoV (Fig. 1e). We noted that most of the TFs that are differentially expressed and shared between two or more of the Coronaviruses infected cells are members of the Znf TF family (ZNF436, 448, 543, 597, 773, XSCAN12, ZEB1, ZDTB20, KLF10, and HIVEP1) bHLH family (MXD1 and MXD4), involved in CCAAT/Enhancer Binding Protein (C/EBP) (DDIT3 and CEPPD), NF-κB complex (RELB), AP-1 complex (FOSB, JUN), ETS family (SPDEF) and E2F TF, among others (Supplementary Fig. 1c).

Since the repeated elements contained in the genome and their expression can also be regulated by epigenetic components, we evaluated the changes in gene expression of repeat elements after viral infection (Supplementary Table 3). We found that 47, 22, and 319 repeat elements are differentially expressed in SARS-CoV-2, SARS-CoV, and MERS-CoV infected cell lines, respectively. In SARS-CoV-2, the repeat elements belong predominantly to the long interspersed nuclear elements (LINE; 13 elements), long terminal repeat (LTR; 17 elements), and DNA repeat elements (17 elements) families. Similarly, for SARS-CoV and MERS-CoV we found LINE (5 and 59 elements), LTR (10 and 179 elements), and DNA repeat (5 and 65 elements). Interestingly, short interspersed nuclear elements (SINE) elements are not differentially expressed (only three elements found in MERS-CoV) and few Satellite elements were identified (3, 1, and 10 in SARS-CoV-2, SARS-CoV, and MERS-CoV, respectively) The elements L1MA4:L1:LINE, L1PA8A:L1:LINE and LTR54:ERV1:LTR are shared among all viruses. Notably, the L1 or LINE-1 elements are the only autonomous transposons that remain active in the human genome and are mainly repressed by epigenetic mechanisms such as HPTMs (H3K9me3)^20^. The latter, along with the fact that SARS-CoV-2 infected cells overexpress the histone demethylases KDM7A and KDM6B that target the heterochromatin histone marks such as H3K9me and H3K27me^21^ (Supplementary Fig. 1c), suggest that SARS-CoV-2 infection could promote an open chromatin conformation, thus affecting the transcriptional expression and the derepression of the repeated sequences.

### SARS-CoV-2 transcriptional effect in COVID-19 patient-derived samples

**Fig. 2:**
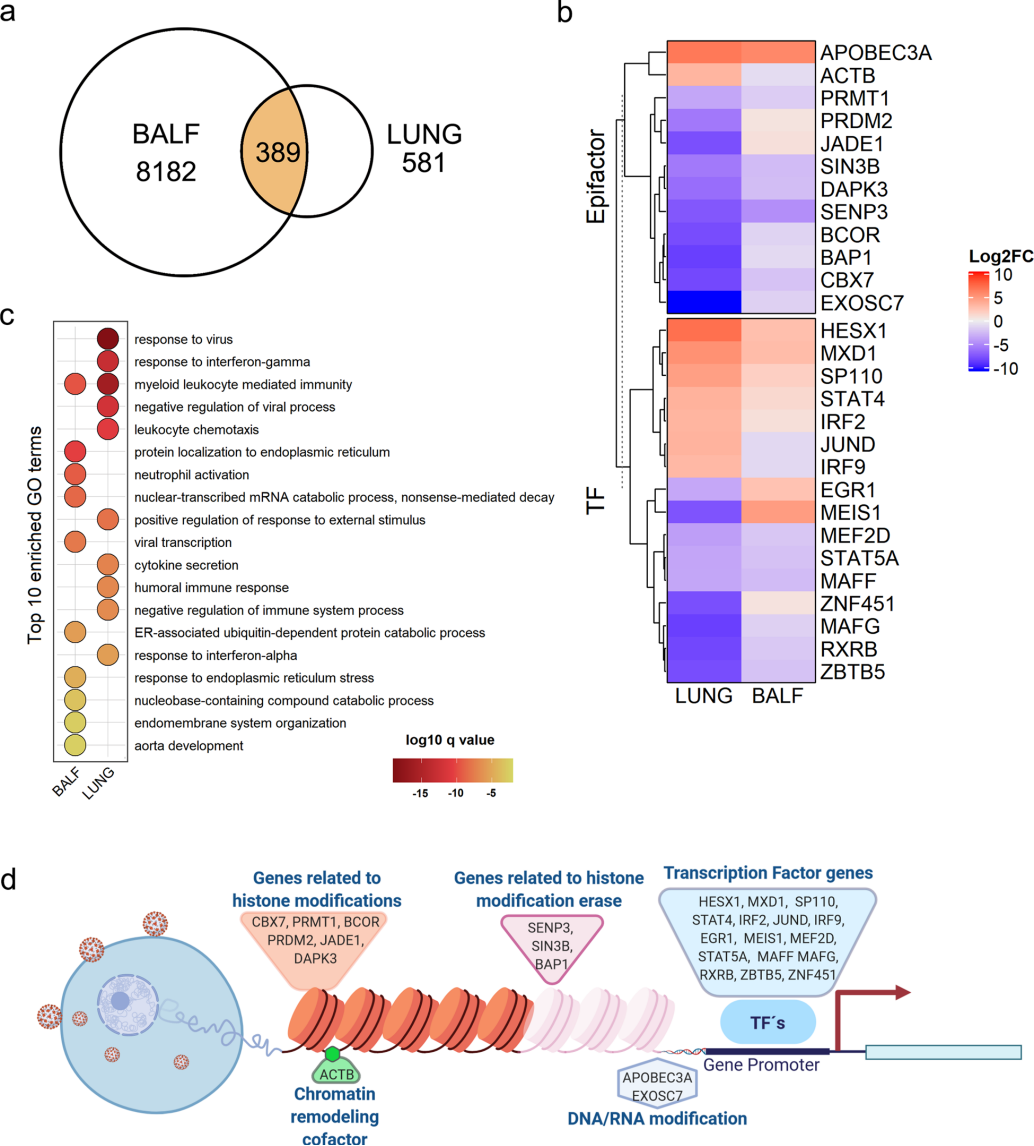
Differential expression analysis of COVID-19 patient samples. **a** Number of shared differentially expressed genes between the samples. **b** Log2 fold change of shared differentially expressed epigenes in patients’ samples. **c** Top ten simplified Gene Ontology enriched terms belonging to the biological process sub-ontology; ordered by *q*-value. **d** Epigenetic processes associated with the shared differentially expressed epigenes between patient samples. Created with BioRender.com.

Afterward, we evaluated the transcriptional response in patient samples infected with SARS-CoV-2 to assess their resemblance to the previously observed results in cell lines. For this purpose, we obtained datasets from samples of bronchoalveolar lavage fluid (BALF) and lung. From the differential expression analysis, we found 389 DEGs shared among both samples (Fig. 2a). In this geneset, we identified 28 epigenes whose fold change direction was consistent in most of the cases (Fig. 2b); GO enrichment analysis shows that most of the DEGs were related to the immune response to viral infection such as leukocyte mediated immunity and humoral immune response (Fig. 2c). Furthermore, they were involved in a wide range of epigenetic processes, such as histone modification, chromatin remodeling, DNA modification, and TF (Fig. 2d). Following, we evaluated the similarity of these results with the data observed for SARS-CoV-2 in infected cell lines by comparing the overlap between the virus-associated genes with the DEGs present in the patient’s samples (hereinafter referred as patient-DEGs, 389 genes). We found 46, 10, and 22 genes in common with SARS-CoV-2, SARS-CoV, and MERS-CoV respectively. In particular, for SARS-CoV-2 infected cell lines and patients, 5 TFs (*STAT5A, MAFF*, *IRF9*, *MXD1*, and *STAT4)* and no epifactors were identified. The shared DEG between samples of SARS-CoV-2 infected patients and MERS-CoV and SARS-CoV infected cell lines, which were found at a lesser extent, is likely to be non-specific viral-responding immune genes. Finally, we found that 804 and 20 repeat elements are differentially expressed in BALF and LUNG samples, respectively, being LTR elements the most differentially expressed in both samples (Supplementary Table 3). Collectively, these results show that the gene expression changes promoted by SARS-CoV-2 infection in patients are similar in respiratory tract samples, where immune response processes are the main ones affected.

### SARS-CoV-2 and MERS-CoV infection induce different transcriptional fold changes in shared gene co-expression modules, which recapitulate the expression profiles in COVID-19 patient-derived samples

So far, our analyses showed that cell lines and patient samples infected with SARS-CoV-2 exhibited DEGs related to immunological processes, which has been previously described by Blanco-Melo et al.^22^ and is congruent with our results. However, differential expression analysis often overlooks the subtle differences in several genes that all together can be responsible for major changes in global transcriptional regulation. Weighted gene co-expression network analysis overcomes this limitation by studying the expression of thousands of genes in the same analysis^23^. Thus, we expanded our previous results by including a co-expression analysis to identify gene modules associated with each viral infection and genes that play central roles within them.

**Fig. 3:**
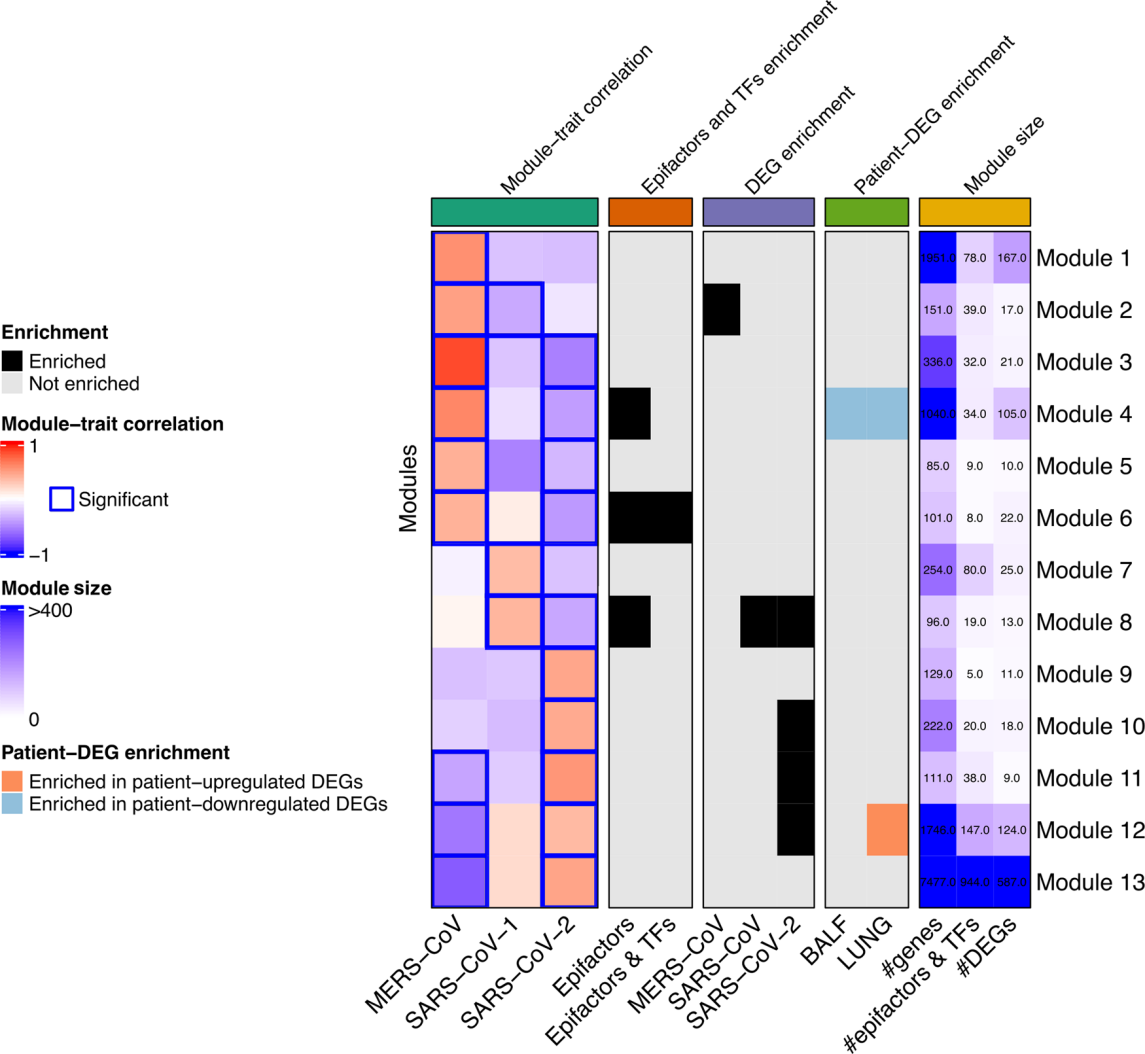
Relevant modules for coronavirus infection. Summary of the analyses used to identify relevant modules for each infection. From left to right, grids show the module-trait correlation, the enrichment of epigenes, the enrichment of DEGs found in cell lines, enrichment of DEGs found in patients’ samples, and information of the module size.

We constructed the co-expression network with the log2 fold changes of each sample compared to its controls. After identifying the modules, we calculated the correlation between each module and the different traits, where we found that out of the 24 total modules identified, 13 were significantly correlated to the infection of any of the three viruses (Supplementary Fig. 2; Supplementary Table 4). Specific modules are associated with MERS-CoV (module 1), SARS-CoV (module 7), and SARS-CoV-2 (modules 9 and 10) (Fig. 3); shared modules were also identified. Notably, more than half of the modules (7 out of 13) are significantly associated with both SARS-CoV-2 and MERS-CoV; contrary to SARS-CoV-2 and SARS-CoV, which have only one module jointly associated. Remarkably, SARS-CoV-2 and MERS-CoV share a higher number of modules that are significantly associated with each virus. Even though the transcriptional profile is not the same, as it can be seen by the opposing response, the same genes from the shared modules are involved in both infections. Therefore, those infections share more players relevant to the infection than SARS-CoV-2 and SARS-CoV, as it would be expected.

Further analysis with GO enrichment analysis (Supplementary Fig. 3), shows that genes in modules 11 and 12 are involved in the host cell response to viral infection, while modules 4, 5, 7, 8, 10, and 13 were associated with intracellular processes used by the viruses during the infection, such as DNA replication, translation, ribosome biogenesis, and protein folding. Interestingly, module 6 was found related to epigenetic processes, particularly, transcriptional activation of promoters. These results show that, in addition to the immunological processes identified in our previous differential expression analysis, the transcriptional response to SARS-CoV-2 and MERS-CoV infection contain genes that also participate in RNA translation, DNA replication, and epigenetic regulation.

Following, in order to determine the modules that might be more relevant to SARS-CoV, MERS-CoV, or SARS-CoV-2 infection in terms of epigenetic regulation, we conducted enrichment analyses to identify an over-representation of epigenes, virus-associated gene sets, DEGs found in patients (Fig. 3).

When integrating these analyses, we found module 1 relevant for MERS-CoV and module 7 for SARS-CoV infection since they are exclusive for these viruses according to the co-expression analysis (Fig. 3). To determine the most important epigenes for each of these modules, we evaluated the eigengene-based connectivity (Module Membership, MM) to find hub genes and the gene significance (GS) of each gene. Subsequently, we prioritized them by identifying drugs targeting them. After looking for candidate drugs that targeted these central players, we found approved medication for CDK7 and PCNA for SARS-CoV and NCOA1, NR1H2, PRKAB2, CLOCK, KDM1B, and ATF2 (Supplementary Fig. 4).

Regarding SARS-CoV-2, module 9 was uniquely associated with it, but also other modules stood out. First, module 4 had a consistent behavior between cell lines and patients, since we found it negatively correlated to SARS-CoV-2 while being enriched in downregulated genes in BALF and LUNG samples; in addition, it was enriched in epifactors, suggesting an important role in viral-related epigenetic modifications carried out by these genes. A similar phenomenon is observed for module 12, which was positively correlated with SARS-CoV-2 infection and enriched with upregulated genes in patients and with SARS-CoV-2 DEGs. In addition, modules 10 and 11 were positively correlated to SARS-CoV-2 and enriched in SARS-CoV-2 DEGs, with module 11 being also enriched with patient’s DEGs. Module 6 was negatively associated with SARS-CoV-2 in the co-expression network and enriched with epigenes, and module 8 was enriched with upregulated genes in PBMC and negatively associated with SARS-CoV-2 in the co-expression network while being enriched with epifactors and SARS-CoV-2-DEGs (Fig. 3). Finally, the enrichment of TFs targets in each module was evaluated to identify the ones that could explain the co-expression patterns of the genes within the module. With this analysis, it was found that Module 4 is enriched in the target genes of the transcriptional factors MTA1, MORC2, and RBM34 that belong to the same module (Supplementary Table 5), being MTA1 and RBMC34 differentially expressed in BALF samples. It is worth mentioning that in most of these modules epigenes showed a higher MM than the rest of the genes (Module 1: *W* = 164,249, *p* < 0.05, Module 2: *W* = 1497, *p* < 0.05, Module 4: *W* = 51,768, *p* < 0.05, Module 6: *W* = 1359, *p* < 0.05, Module 7: *W* = 3741, *p* < 0.05, Module 12: *W* = 113,820, *p* < 0.05, Module 13: *W* = 2,182,049, *p* < 0.05), evincing their central role within their modules.

Collectively, these results show that the transcriptional response to the infection of SARS-CoV-2 and MERS-CoV involve a higher similarity regarding gene modules but with a different extent of transcriptional change in host cells during infection, which extends our previous observations in the differential expression analysis. Therefore, the same genes in the shared modules play a potential role in both infections, despite presenting a different transcriptional behavior. Importantly, the virus-correlated co-expression modules either recapitulate the changes in gene expression observed in different COVID-19 patient sample types or are enriched with epifactors, and also contain genes involved in several biological processes related to viral infection, suggesting that the data obtained in the cell lines could recapitulate what was found in infected patients

### Protein–protein interaction network analysis provides additional therapeutic alternatives and new targets for drug development for COVID-19

To prioritize epigenes that play a key function in each co-expression module relevant for SARS-CoV-2 (modules 4, 6, 8–12), we examined them at the protein–protein interaction (PPI) level in the context of SARS-CoV-2 infection. We constructed a PPI network containing all experimentally validated human protein interactions^24^ and the reported virus–host protein interactions from Gordon et al. and Stukalov et al.^25,26^. Using the virus–human PPI network, we performed de novo pathway enrichment analysis with KeyPathwayMiner^27^ to extract the largest network using a selection of epigenes as input, while also taking into account the SARS-CoV-2-DEGs and patient–DEGs previously identified. The selection of epigenes for each module was based on their shortest path length with viral proteins, their expression correlation with viral genes, and their MM.

**Fig. 4:**
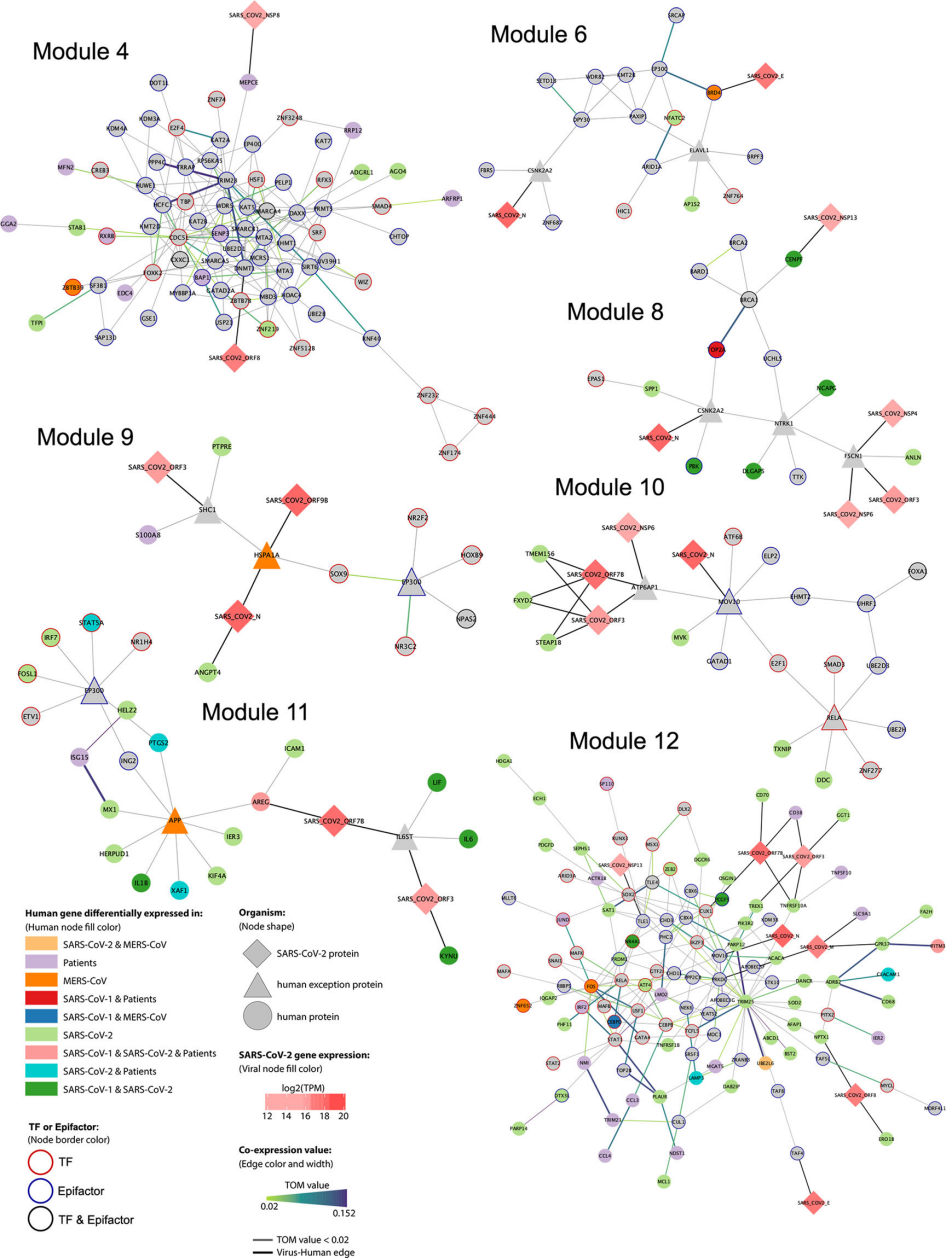
Protein-protein interactions network containing SARS-CoV-2-DEGs, patient-DEGs, or selected epigenes for modules 4, 6, 8–12. Nodes and edges represent proteins and the interaction between them, respectively. The node and edge color’s meaning is indicated in the annotation panel.

All genes contained in the networks identified (Fig. 4) provide insights about the molecular machinery involved in SARS-CoV-2 infection, since the genes are either differentially expressed in infected cell lines or patients, or they are hub-epigenes in the co-expression analysis.

For module 4, the network obtained contains mainly epifactors. Notably, DNMT1 directly interacts with the viral protein ORF8 and with TRIM28, to which it is also highly co-expressed. Other relevant epigenes in the networks are SIRT6 (highly co-expressed and interactor of TRIM28), SENP3, MTA1 (a TF whose targets are also enriched in module 4; Supplementary Table V), and BAP1 (differentially expressed in patients). Furthermore, MEPCE, an snRNA methyl phosphate capping enzyme, is differentially expressed in patients and interacts with viral protein NSP8. Module 6 contains BRD4, which directly interacts with E viral protein and is highly co-expressed with EP300, a histone acetyltransferase. Another relevant epigene is SETD1B (related to the trimethylation of H3K4^3^, a unique epigenetic histone mark related to transcriptional activation), which interacts with TRIM28, present in module 4^28^. For module 8, notable epigenes are CENPF, differentially expressed in SARS-CoV and SARS-CoV-2 and directly interacting with NSP13; and TOP2A, differentially expressed in SARS-CoV infected cell lines and patients. EP300, an exception node in module 9, interacts with several TFs such as NR2F2, HOXB9, NR3C2, and SOX9. In module 10, RELA (also known as nuclear factor NF-κB p65 subunit) and MOV10 are exception nodes that interact with the TFs SMAD3, ZNF277, and UBE2D3. Also, viral proteins ORF7B and ORF3 interact with FXYD2, STEAP1B, and TMEM156, which are differentially expressed in SARS-CoV-2 infected cell lines. For module 11, IRF7 (interferon regulatory factor 7) and STAT5A interact with EP300. Module 12, also contains epigenes of interest, such as MOV10 (Putative helicase MOV-10) that interacts with the N protein, TRIM25, RELA, and TLE1, which has a direct interaction with viral protein NSP13. Further genes which are classified as hub-epigenes and are also differentially expressed in cell lines or patients are FOS, CEBPD, NR4A1, PRDM1, PCGF5, ZNF652, IRF2, and ZEB2.

Briefly, we identified relevant TFs known to participate in Coronavirus infection and support the veracity of our results, such as TFs from the STAT family (STAT1, STAT2, and STAT5A), interferon regulatory factors (IRF7 and IRF2), cytokines (CCL3, CCL4, and IL1B), and FOS and JUND, members of the AP-1 complex^29^. However, we also identify important genes that appear to be drivers of SARS-CoV-2 infection; such as the epifactors MOV10 and EP300 and the TF RELA, since they are exception connectors (genes that do not belong to the specific module, but are important in the protein pathway found) in more than one module (modules 6 and 9–11), and belong to modules enriched in genes that participate in histone H3–K4 methylation and in the response to interferon-gamma. EP300 is a histone acetyltransferase that was also identified in SARS-CoV-2 infected cell lines^14^. Additionally, we found that MOV10, a putative helicase, also participates in SARS-CoV-2 infection. The TF RELA has been increasingly recognized as a crucial modulator of the response to SARS-CoV-2 infection^14,30^ and is part of the NF-κB complex, along with RELB^31^, which is differentially expressed in MERS-CoV and SARS-CoV-2 infected cell lines (Supplementary Fig. 1D). TRIM25 is a ubiquitin ligase required for the production of INF-1 and is inhibited by the Nucleocapsid of SARS-CoV^32^. Finally, TRIM28 (also known as KAP1) has been shown to interfere with viral integration into host genome^33^ and represses the expression of repeat elements of the LINE family, in particular L1NA4^21^, which was previously identified as differentially expressed in cell lines infected with the three Coronaviruses.

Afterward, we evaluated whether the proteins in the networks had annotated drugs targeting them. We found drugs for 69 out of the total 260 proteins, being PLAU, RELA, NEK6, NR1H4, PTGS2, PRKDC, ESR1, NR3C2, TTK, TOP2A, ADRB2, HDAC4, TRIM25, STK10, RPS6KA5, and EP300 the ones with the most drugs identified (more than 20). A total of 799 drugs were found, where Erlotinib, Imatinib, Lapatinib, Sunitinib, S-adenosyl-L-homocysteine (SAH), Quercetin, Tandutinib, RAF-265, Pictilisib, Neratinib, and Fedratinib are the drugs with more targets (more than 5; Supplementary Table 6). Relevant epigenes that have associated medication are shown in Table 1.

**Table 1:** Drugs targeting candidate epigenes from modules 4, 6, 8, and 10–12.

Target protein	Drug name	Module	Function
TOP2A	Genistein, fluorouracil, intoplicine, enoxacin, sparfloxacin, amrubicin, etoposide, epirubicin, ciprofloxacin, myricetin, mitoxantrone, trovafloxacin, RTA 744, daunorubicin, norfloxacin, finafloxacin, dexrazoxane, 13-deoxydoxorubicin, idarubicin, lomefloxacin, lucanthone, pefloxacin, valrubicin, amsacrine, levofloxacin, doxorubicin, declopramide, annamycin, banoxantrone, ZEN-012, podofilox, aldoxorubicin, teniposide, moxifloxacin, SP1049C, amonafide, dactinomycin, fleroxacin, becatecarin, ofloxacin, and elsamitrucin	Module 8	Epifactor (chromatin remodeling)
BRD4	Fedratinib, panobinostat, romidepsin, birabresib, alprazolam, vorinostat, volasertib, alobresib, belinostat, and apabetalone	Module 6	Epifactor (histone modification read)
EP300	Curcumin	Module 6	Epifactor (histone modification write)
DNMT1	S-adenosyl-L-homocysteine, procainamide, palifosfamide, cefalotin, decitabine, azacitidine, flucytosine, epigallocatechin gallate, and hydralazine	Module 4	Epifactor (DNA methylation)
SENP3	Methylphenidate	Module 4	Epifactor (histone modification erase, histone modification write cofactor)
SIRT6	7-[4-(Dimethylamino)Phenyl]-N-Hydroxy-4,6-Dimethyl-7-Oxo-2,4-Heptadienamide	Module 4	Epifactor (histone modification erase)
FOS	Pseudoephedrine and nadroparin	Module 12	TF
RELA	SC-236, betulinic acid, bortezomib, dimethyl fumarate, PHENYL-5-(1H-PYRAZOL-3-YL)-1,3-thiazole, indoprofen	Module 12	TF
STAT1	Epigallocatechin gallate	Module 12	TF
STAT5A	AZD-1480	Module 11	TF
SMAD3	Ellagic acid	Module 10	TF

Most notably, RELA is targeted by SC-236, bortezomib, indoprofen (an anti-inflammatory) and betulinic acid, whose derivatives show anti-HIV activity^34,35^. EP300 is targeted by curcumin, a molecule with anti-inflammatory properties^36^. The latter proposes RELA and EP300 as new potential drug target candidates for SARS-CoV-2 infection, not only because they participate in immune-related processes, but also because they belong to the cellular epigenetic machinery used by the virus during infection. Furthermore, self-evident immune-related targets STAT5A, STAT1, and FOXN2 are also good candidates for treatment. Finally, the proteins MOV10, TRIM25, and TRIM28 do not have associated drugs, thus they are good candidates for drug development, as well as other relevant epigenes shown in Table 2.

**Table 2:** Candidate epigenes for drug development in modules 4, 6, 8, and 10–12.

Target protein	Module	Function
MOV10	Module 12	Epifactor (chromatin remodeling)
MTA1	Module 4	Epifactor (chromatin remodeling cofactor)
TLE1	Module 12	Epifactor (chromatin remodeling, histone modification cofactor)
TAF4	Module 12	Epifactor (histone chaperone)
BAP1	Module 4	Epifactor (histone modification erase, polycomb group (PcG) protein)
TRIM28	Module 4	Epifactor (histone modification read)
PRDM1	Module 12	Epifactor (histone modification write cofactor)
SETD1B	Module 6	Epifactor (histone modification write)
UBE2D3	Module 10	Epifactor (histone modification write)
PCGF5	Module 12	Epifactor (polycomb group (PcG) protein)
STAT2	Module 12	TF
GMEB2	Module 6	TF
ZNF277	Module 10	TF
IRF7	Module 11	TF
CEBPD	Module 12	TF
NR4A1	Module 12	TF
ZNF652	Module 12	TF
IRF2	Module 12	TF
ZEB2	Module 12	TF
SP110	Module 12	TF
CUX1	Module 12	TF
TRIM25	Module 12	E3 ubiquitin ligase

**Fig. 5:**
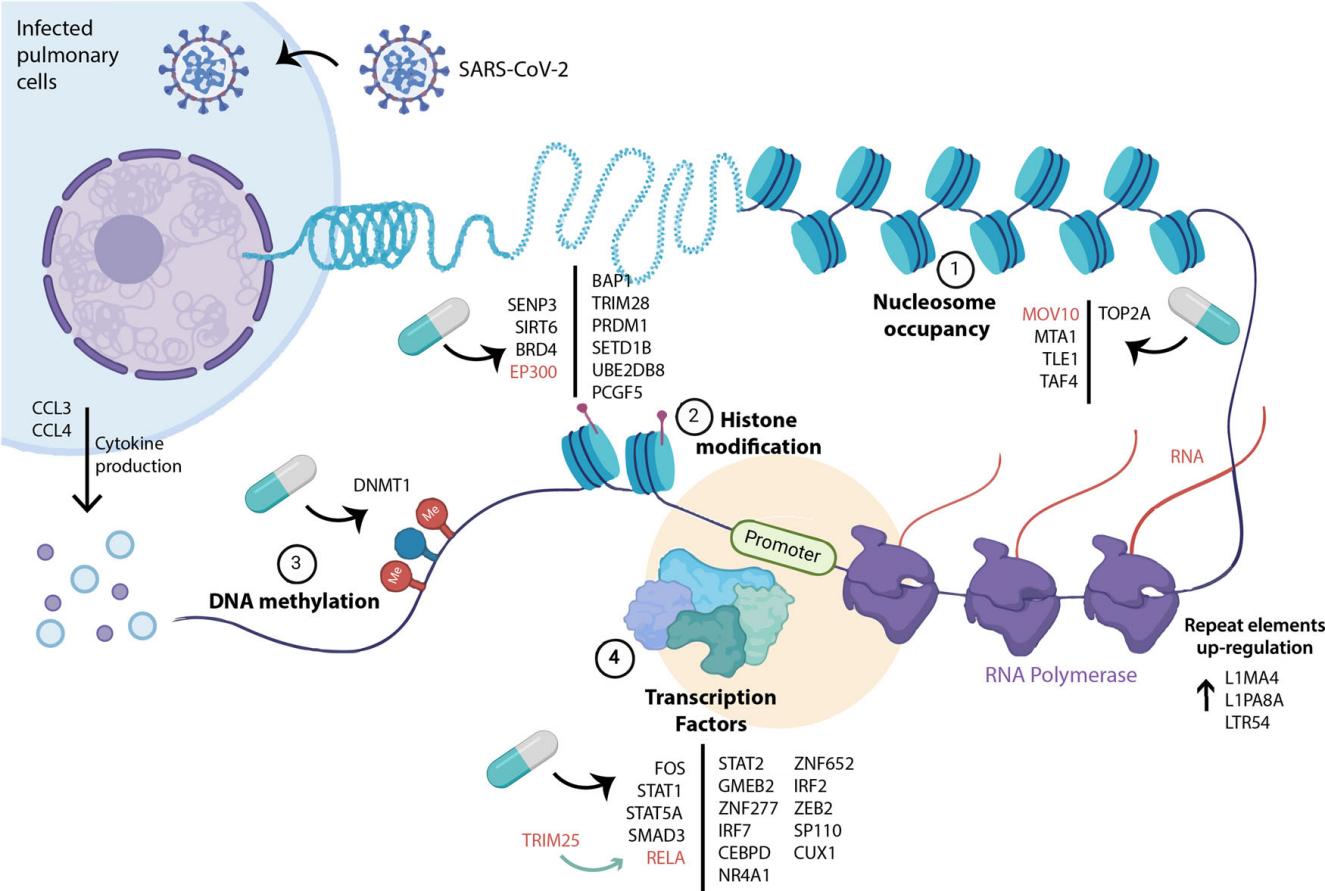
Relevant epigenes in SARS-CoV-2 infection with therapeutic potential. Epigenetic targets are indicated in different processes such as nucleosome occupancy (1), histone modification (2), DNA methylation (3), and also TFs (4). Top gene candidate targets are highlighted in red. Created with BioRender.com.

Together, network analysis at the protein level allowed the identification of several epigenes that are part of the molecular machinery used by the virus during infection (Fig. 5). Epigenes that participate in the immune response through different mechanisms (response to interferon or NF-kB complex) are among the main genes identified and are evident drug target candidates for COVID-19 because they already have associated drugs targeting them (such as STAT5A and STAT1). Furthermore, new candidate druggable epigenes were also identified, notable examples are EP300 and RELA, which are targeted by drugs with anti-inflammatory or antiviral properties; and TRIM25, TRIM28, and MOV10, which are good candidates for drug development.

## Discussion

Cells are in constant adaptation with their environment, in fact they can sense and respond to different stimuli by changing their transcriptional patterns. This cellular plasticity allows cells to adapt almost immediately to insults, including virus infections^37^. Epigenetic proteins and TFs are one of the main elements involved in the transcriptional response of cells during viral infection. These elements can be used as protein targets for drug identification and treatment. In this work, we aimed to identify key TFs and proteins involved in the epigenetic response to viral infection of SARS-CoV-2, SARS-CoV, and MERS-CoV by integrating co-expression and de novo pathway enrichment analyses. Therefore, our study focused on the infection part of COVID-19, which is relevant mostly during the early stages of the disease, in contrast to the immune pathologies seen in the later ones.

One of our main findings is that the transcriptional response (regarding DEGs and significantly co-expressed modules) induced by SARS-CoV-2 and MERS-CoV involves a higher similarity regarding gene players and biological processes than SARS-CoV-2 and SARS-CoV, despite presenting a different transcriptional behavior. However, it is interesting to notice that regarding the transcriptional trend of the modules (i.e., correlation sign), SARS-CoV-2 and SARS-CoV behave more similarly despite many modules not being significantly associated with SARS-CoV. Nevertheless, unique modules, patterns and DEG were found in each CoV. Despite they belong to the coronavirus family, each one has unique characteristics that could influence its pathogenicity and virulence. This finding agrees with a recent study that has found specific biological process deregulations in SARS-CoV-2 infected cell lines, which are not found in other CoVs^15^. In addition, different transcriptional change patterns have been observed between MERS-CoV and SARS-CoV during the infection; these changes are not recapitulated by phylogenetic relationships since, in some groups of genes, MERS-CoV-infected transcriptional behavior appears to be more similar to the more remotely related influenza H5N1 virus infection^13^.

Furthermore, the contrasting transcriptional response induced by the infection of SARS-CoV-2 and MERS-CoV in several modules suggests that genes in those modules participate in both viral infections but with a different mechanism, which leads to distinct pathways of infection that could explain the dissimilar phenotypes observed in both diseases. Divergent fold change trends, such as the ones described in this study, have been previously described in MERS-CoV and SARS-CoV infections to limit the host type I interferon (IFN-I) response, where predominant active and repressive epigenetic marks in involved genes are the opposite between both CoVs^13^. In our study, we present a list of epigenes and biological processes whose fold change trend is the opposite between MERS-CoV and SARS-CoV-2; further investigation on them could shed light on the mechanisms responsible for the differences in pathogenesis and outcome of both viral infections.

We further identify at the protein interaction level, that several TFs take part mainly in the immunological response to viral infection. One example is NF-κB, whose p65 subunit (also known as *RELA*) is a central part in the protein interaction network for SARS-CoV-2. NF-κB induces the expression of several pro-inflammatory cytokines, including IL-6, CCL2, and CCL3^38^, which had been found in high levels in COVID19 patients^39^. On the other hand, TRIM25, a ubiquitinase, is essential for the activation of NF-κB and the production of IL-6^40^. TRIM25 is overexpressed in cell lines infected with SARS-CoV-2 but not in those with MERS-CoV, which furthermore suggests that NF-κB could be a medullary part of the host immune response against SARS-CoV-2. The previous observation is reinforced by the fact that it was observed that RELA directly interacts with histone acetyltransferase EP300, and both proteins interact with various components of the AP-1 complex such as FOS, JUND, and FOSL1. AP-1. EP300 and NF-κB regulate chromatin accessibility in the proximal promoter region of IL-6 and CCL2, both pro-inflammatory cytokines^41,42^. The p300/CBP complex is one of the best-characterized cofactors of NF-κB and specifically binds RELA and acetylates it along with the surrounding histones^31^. It is known that adults older than 65 years have higher NF-κB levels compared to younger adults^43^ and some authors had suggested that this may be one reason older adults are more susceptible to develop the severe form of COVID-19^44^.

According to our results, SARS-CoV-2 infection modifies the expression of several TFs of the interferon regulatory factor (IRF) and STAT families, which are primarily involved in the immune response against pathogens. STAT1 and STAT2 are key elements of the signaling induced by type I interferons, these proteins form a dimer upon interferon-mediated phosphorylation and, together with IRF9, form the complex ISGF3 that activates the transcription of interferon-stimulated genes^45^. Our results also showed that IRF9 is upregulated in cell lines infected with SARS-CoV-2; however, module 12’s interactome showed that STAT2 and STAT1 interact with IRF2. IRF2 is a negative regulator of IFNα and its inhibition causes an increase in the antiviral response induced by IFNα^46^. This fact further suggests an impairment of interferon type I stimulated genes activation, as previously described as a hallmark of SAR-CoV-2 infection^47^. On the other hand, IRF1 and IRF7 were also upregulated in SARS-CoV-2 infected cell lines. IRF7 is a key TF for IFNα expression, and it has been previously identified as a hub gene for SARS-CoV-2 infection together with IFR9 and STAT1^48^. It is also interesting that IRF7 loss of function mutations was associated with severe COVID-19 patients^49^ and with the development of life-threatening influenza in children^50^ which suggests that inhibition of IRF7 activity is crucial for SARS-CoV-2 pathology.

Viruses have been reported to use epigenetic machinery to take advantage of the cell and hijack its regulatory capacity for their own benefit^13^. The epigenetic machinery can be affected by coronaviruses in this same sense, and this can happen either by promoting alterations in the epigenetic code, such as DNA methylation and post-translational modifications of histones, or directly by promoting the dysregulation of enzymes and other proteins associated with the epigenome.

We found that among the deregulated epifactors with histone acetylation function are HDAC9 and SIRT1 enzymes. In this sense, it has recently been reported that the SIRT1 protein (a class 3 HDAC) was positively regulated in the lung of patients with severe COVID-19 comorbidities^51^. Likewise, another work demonstrated that under conditions of cellular energy stress, SIRT1 can epigenetically regulate the ACE2 receptor^52^. Also, it has been observed that treatment with non-steroidal anti-inflammatory drugs can inhibit SIRT1 activity, which in turn could affect ACE2 expression^53^. Accordingly, it has been postulated that in some diseases where the epigenetic dysregulation is implicit (such as lupus) the entrance of SARS-CoV-2 into the host cells may be facilitated^54^.

Interestingly, the enzymes HAT1, HDAC2, and KDM5B have been reported to also potentially regulate ACE2 in human lungs. KDM5B has gained interest, because it is associated with other viral infections such as the hepatitis B virus^55^, and potentially with SARS-CoV-2^51^. Remarkably, in breast cancer cells, it has been shown that inhibition of this enzyme triggers a robust interferon response that results in resistance to infection by DNA and RNA viruses^56^. In this regard, we observed several deregulated KDMs in the different coronavirus infections, in which KDM6B stands out by being deregulated in both MERS-CoV and SARS-CoV2 infection. KDM6B is a specific demethylase of H3K27me3, which acts as a repressive histone mark. Although it remains to be fully studied, it is associated with the regulation of a wide range of genes involved in inflammatory agents, development, cancer, viral infection response, senescence and is an important host response against environmental, cellular stress^57^. Therefore, adding to the above, it is suggested that demethylases, such as KDM6B, are potential epigenes that are affected during SARS-CoV-2 infection and can be presented as potential targets for the treatment of COVID-19. However, this should be further studied.

Several epigenes previously involved in response to viral infections stood out in our protein interaction analysis, such as BRD4, TOP2A, and TRIM28. Bromodomain protein 4 (BRD4) is a histone acetylation reader and writer that plays an important role in DNA replication, transcription, and DNA repair^58^. This epigene is critical for the maintenance of the higher-order chromatin structure since its inhibition leads to chromatin decondensation and fragmentation, and it also can stimulate innate antiviral immunity^58^. BRD4 complexes with RELA and CDK9 and are functionally required for effective activation of NF-kB-dependent immediate-early cytokine genes in response to viral patterns. In this sense, our results show a PPI with EP300, which involves the p300/CBP complex, one of the best-characterized cofactors of NF-kB and binds specifically to RELA^59^, validating the possible importance of this system in infection with SARS-CoV2. Examples like this suggest that the virus, through these epigenetic remodelers, promotes chromatin remodeling that could lead to opening, both at the local and global level. Accordingly, an indicator of global changes is the increased expression of transcripts from repeated sequences such as LINE1. If this is so, then the virus is manipulating the chromatin aperture to promote the expression of genes that support its invasion. In this regard, other work has suggested the importance of LINE1 elements. Where these types of repetitive elements are very relevant in gene regulation, especially when these elements are in proximity to neighboring genes since they could alter their expression. Therefore, the dysregulation of repeated elements such as LINE1 could indirectly change the cellular transcriptome^60^.

Furthermore, we find epigenes that interact with the viral proteins directly or very closely. This connection suggests a virus-promoted modulation to affect the epigenome of the host cell’s interactome. Which reinforces the idea that the virus strategy is partly to take advantage of the epigenetic machinery. In general, our data suggest that the SARS-CoV-2 infection deregulates the epigenetic master machinery of the host cell. One of the points that should be taken into consideration in the future is that if this epigenetic machinery is not re-established after disease courses it could generate other diseases such as cancer in the long term. This is based on the fact that many of the genes that we found in our study have been proposed as epigenetic hallmarks in various neoplasms.

Our last key finding is the identification of driver epigenetic proteins and TFs involved in SARS-CoV-2 infection that can be targeted by existing drugs. We identified SAH targeting several epigenetic components of the host response to SARS-CoV-2 infection. SAH is the product of the chemical reaction performed by methyltransferases using nucleic acids or proteins as substrates and has been previously suggested as a potential treatment for viral infections such as ZIKA, MERS-CoV, and SARS-CoV^61–64^ due to its inhibitory activity of the viral RNA cap 2′-O-methyltransferase, formed by the NSP16-NSP10 complex^65,66^. Furthermore, given the interaction between DNMT1 and ORF8 at the protein level, SAH could potentially work against SARS-CoV-2 infection, not only by inhibiting the methyltransferase activity of NSP16–NSP10 but also by directly modulating the activity of the key host proteins involved in the transcriptional response to infection or by interfering with the interactions observed between ORF8 and DNMT1.

Furthermore, as anticipated, many proteins with epigenetic functions involved in SARS-CoV-2 infection have kinase activity and can be targeted by kinase inhibitors. One important example is imatinib, which we identified as a potential drug for SARS-CoV-2 and SARS-CoV, and is currently undergoing clinical trials to evaluate its efficacy in COVID-19 patients (NCT04394416, NCT04422678, NCT04346147, and NCT04357613; www.clinicaltrials.gov). Similarly, we found quercetin targeting several epifactors with kinase activity. Quercetin is a plant-derived compound with anti-inflammatory and antiviral effects^67,68^ that has been evaluated in clinical trials as a dietary supplement or prophylaxis for COVID-19 (NCT04578158, NCT04377789, and NCT0446813). Even though some independent studies show no clear evidence of its effectiveness, preliminary data shows that it could be effective to decrease the frequency and duration of respiratory tract infections^69–71^. It is worth mentioning that these drugs are being tested in clinical trials based on their described inhibitory activity of enzymes related to the activation of immune response and inflammation, such as growth receptors^72^. The latter, together with our results, suggests that drugs targeting epigenetic mechanisms could be also effective to treat SARS-CoV-2 by modulating their kinase activity.

Finally, we also identified Bortezomib and betulinic acid associated with RELA. Bortezomib is a proteasome inhibitor that has been proposed as COVID-19 therapy given its capacity to inhibit (although only marginally) the papain-like protease (NSP3) of SARS-CoV, which also has deubiquitinase activity^73–75^. Likewise, betulinic acid has been proposed as a target of NSP3 in SARS-CoV-2^76^.

Together, we have supporting evidence that current drug-based therapies to treat COVID-19 also target the transcriptional response to infection by the modulation of the epigenetic proteins identified in this study. Furthermore, we provide additional new potential drug targets and drug candidates which could be effective and whose potential use has not been exploited yet. These results provide comprehensive evidence that epigenetic therapy could aid in restoring the transcriptional changes observed during infection. By using epigenetic drugs, a therapeutic effect can be achieved due to their systemic effects, which can be advantageous to treat a disease that targets different tissues and cellular mechanisms, as observed in COVID-19.

In this study, we used a blend of bioinformatic approaches to comparatively analyze transcriptomic data from SARS-CoV-2, SARS-CoV, and MERS-CoV infected pulmonary cell lines and COVID-19 patient-derived samples. In particular, we focused on the epigenetic processes and transcriptional factors, since these have been widely proposed as the master regulators of the expression of most genes. We found that the transcriptional response to the infection of SARS-CoV-2 and MERS-CoV is more similar to that observed for SARS-CoV regarding shared significantly associated gene modules; however, the transcriptional change elicited by MERS-CoV and SARS-CoV seems to be opposite. At the same time, we identified specific altered modules in the response to infection with SARS-CoV2 that could serve as a guide for the proposal of different therapeutic strategies based on epigenetic therapy. Thus, our results add a piece to the puzzle of the strategies used by the different coronaviruses to manipulate the gene regulation capacity of the cell. Although the pathways are differential between them, the virus’s objective is to take advantage of the TFs and various chromatin remodelers to avoid being detected and prevail in the invasion. This is a very fine strategy that the virus uses and it has been poorly studied in both its biological importance and its future therapeutic application. This could open a new window of opportunities for treatment and thus close the chapter on this pandemic disease.

## Methods

### Data processing and differential expression analysis

Raw sequencing data was trimmed with Trimmomatic version 0.39^77^ using the parameters ILLUMINACLIP 2:30:10 LEADING:3 TRAILING:3 SLIDINGWINDOW:4:15 MINLEN:36; and the quality of reads was evaluated with FastQC version 0.11.9^78^. Technical replicates (when existing) were merged and each biological replicate was aligned to the GRCh38 v33 human genome with STAR version 2.7.3^79^ using the mapping parameters suggested in Jin et al.^80^: (—outFilterMultimapNmax 100—winAnchorMultimapNmax 100). To estimate the abundance of the transcripts accounting for coding and non-coding genes as well as repetitive elements, we used TETranscripts version 2.1.4^80^ with the multi-mode. Raw count tables were used for differential expression analysis using DESeq2^81^. DEGs were identified with a p adj. < 0.05 and abs(log_2_ fold change) > log_2_(1.5).

### Viral transcripts quantification

The viral transcriptome was constructed with the 11 gene sequences reported in the SARS-CoV-2 genome (NCBI Reference Sequence NC_045512.2). Viral transcript expression was quantified in each trimmed RNA-seq file of SARS-CoV-2 infected samples with Salmon v 1.3.0^82^.

### Virus and patient DEGs

Virus-associated gene sets were obtained with the intersection of DEGs identified in all the cell lines infected with the corresponding virus, except for SARS-CoV-2. For SARS-CoV, the intersection between the cell lines infected consisted of 182 genes (SARS-CoV-DEGs); for MERS-CoV, the intersection was 1139 genes (MERS-CoV-DEGs); and for SARS-CoV-2, the intersection between at least 3 out of the 4 cell lines was used instead, and consisted in 909 genes (SARS-CoV-2-DEGs) (Supplementary Table 2). The patient-associated gene set was obtained with the shared DEGs in lung and BALF conditions (389 genes, patient-DEGs) (Supplementary Table 2).

### Epigenes catalog

To build the Epigenes catalog, 4 different databases were used: EpiFactors^83^, Histome^84^, dbEM^85^, and the manually curated TF list from Lambert et al.^86^. TFs’ functional annotation was taken from Lambert et al.^86^. The final list consisted of 2161 genes (776 epifactors, 1348 TFs, and 41 categorized as both TF and epifactor).

### Co-expression analysis

Count matrices of the analyzed cell lines were filtered to remove low-expressed genes using the function filterByExpr from edgeR^87^ while accounting for the treatment (i.e., virus infection) and cell type in the filtering design. Following, normalization of gene counts was performed with vst function from DESeq2^81^ (treatment and cell type of each sample were included in the design matrix and accounted for these effects with the blind argument). The gene co-expression network was built with the log_2_ fold changes (log_2_FC) of each biological sample compared with the controls of the same biological condition by applying the formula (1).

1 $$\log _2{\rm{FC}}_i={\mathrm{log}}_{\mathrm{2}}{\mathrm{(}}{\rm{SC}}_i{\mathrm{ / }}{\rm{ACC}}_i{\mathrm{)}}$$

where SC and ACC correspond to the normalized counts of gene *i* in the infected and controls samples, respectively. The resulting matrix containing the log2FoldChanges per sample was used to construct the weighted gene co-expression network with the WGCNA package^23^. A soft threshold of 9 was used to construct the network and modules were identified with a minimum size of 20. Modules whose expression was similar were merged using a dissimilarity threshold of 0.25, resulting in a total of 24 modules. Finally, the module-eigengene Pearson correlation of each module with the viruses was tested.

### Enrichment analysis

Gene Ontology (GO) enrichment analyses were performed using clusterProfiler^88^ in virus-associated and patient gene sets. For the differential expression analyses of infected cell lines, the enrichment of GO terms in DEGs was tested using the expressed genes on each particular comparison as background. For the co-expression network, the enrichment of GO terms was tested in each module using the genes of the full network as background.

Epigenes, virus-associated DEGs, and TF-target enrichment analyses were performed with gProfiler2^89^ using a custom gmt file or the TRANSFAC database included in the package for TF-target enrichment. The correction method used was g:SCS and an adjusted *p* value significance threshold of 0.05. As background, all the genes annotated in the co-expression network were used for epigenes and TF-target enrichment and the expressed genes in each virus for virus-associated DEG enrichment.

### Co-expression module selection

SARS-CoV-2 modules were selected from the co-expression analysis based on whether they were uniquely and significantly associated with SARS-COV-2 in the co-expression analysis. If they were not uniquely associated with SARS-CoV-2, the modules enriched with at least one dataset (DEG, patient–DEG, or Epigenes) were selected. Based on these criteria, modules 4, 6, and 8–12 were selected. MERS-CoV and SARS-CoV modules were selected on whether they were uniquely associated with each specific virus in the co-expression analysis. Module 1 was selected for MERS-CoV and module 7 for SARS-CoV. SARS-CoV-2 selected modules were further analyzed, as described in the following sections.

### Virus–host network construction

Virus–human interactions were obtained from Gordon et al.^25^ and Stukalov et al.^26^. The human PPI network was obtained from IID version 2018-11^24^ using only the experimentally validated interactions (“exp”, “exp;ortho”, “exp;ortho;pred”, or “exp;pred”). After homogenizing the viral protein nomenclature, the three sources of interactions were merged to create the entire virus-human PPI, followed by the removal of duplicated edges and self-loops. The final integrated network contained 30 viral nodes, 17,524 human nodes and 329,054 edges. The mapping of viral transcript counts to viral proteins in the PPI was based on the reference sequence annotation (NCBI Reference Sequence NC_045512.2) and the data provided in Supplementary Data from Gordon et al.^25^.

### Epigene selection

For co-expression modules 4, 6, 8, and 10–12, relevant epigenes were selected based on whether they satisfied at least one of the following criteria: (1) its shortest path length with viral proteins, (2) the correlation value between its expression and the expression of viral proteins, and (3) its MM value, a measure of the correlation between a gene expression profile and the module eigengene, which is highly related to the intramodular connectivity, and GS the correlation of a gene with an external trait (viral infection)^90^.

1. The shortest path length was calculated between all pairs of viral proteins and human proteins in the PPI network with the igraph package version 1.0.0^91^. The retained epigenes were the ones whose shortest path length with at least one viral protein was less than 3.
2. Pearson’s correlation coefficient was computed between the count values of viral transcripts and count values of epigenes in infected cell lines. Epigenes with *p* value < 0.05 and abs(correlation_estimate) > 0.5 with at least one viral transcript were selected.
3. Epigenes with abs(MM) > 0.8 in the corresponding module of the co-expression network were retained.

For modules 1 and 7, epigenes with abs(MM) > 0.8 and abs(GS) > 0.3 were selected.

### De novo pathway enrichment

De novo pathway enrichment analysis for co-expression modules 4, 6, 8, 10–12 was performed with KeyPathwayMiner^27^, the built virus–human PPI network, the full list of viral proteins as positive nodes and a customized input indicator matrix for each module containing as active genes those which belonged to any of the following categories: (1) it was a SARS-CoV-2-DEG, (2) it was a patient-DEG, or (3) it was an epigene selected as described above. The parameters used for all the analyses were the Greedy search algorithm, INES search strategy, remove border exception nodes, *L* = 0, and *K* = 0 for modules 4 and 12, *K* = 2 for module 6, and *K* = 3 for modules 8–11.

### Drug identification

All approved and non-approved drugs targeting the genes/proteins contained in each network were obtained with CoVex^92^ by mapping the gene names to uniprot IDs, using the closeness centrality algorithm and the following parameters: result size = 50,000, disabled hub penalty, disabled max degree, include indirect drugs = FALSE and include non-approved drugs = TRUE. The latter parameters ensure the retrieval of all drugs associated with the input genes. A total of 265 out of 277 genes mapped to the CoVex database.

### Reporting summary

Further information on research design is available in the Nature Research Reporting Summary linked to this article.

## Supplementary information

Supplementary Information

Supplementary Data 1

Supplementary Data 2

Supplementary Data 3

Supplementary Data 4

Supplementary Data 5

Supplementary Data 6

Reporting Summary

## Data Availability

Raw RNA-seq data was obtained from the Sequence Read Archive (www.ncbi.nlm.nih.gov/sra) of the National Center for Biotechnology Information (NCBI), U.S. National Library of Medicine, and the Genome Sequence Archive in BIG Data Center (bigd.big.ac.cn/), Beijing Institute of Genomics (BIG), Chinese Academy of Sciences (Supplementary Table 1).
