# Supplementary material for: Comparative transcriptome analysis reveals key epigenetic targets in SARS-CoV-2 infection
Source: NPJ Syst Biol Appl. 2021 May 24;7:21. doi: 10.1038/s41540-021-00181-x (PMC8144203; doi:10.1038/s41540-021-00181-x)
Supplement: Supplementary file 1 — Supplementary Information [file 41540_2021_181_MOESM1_ESM.pdf]

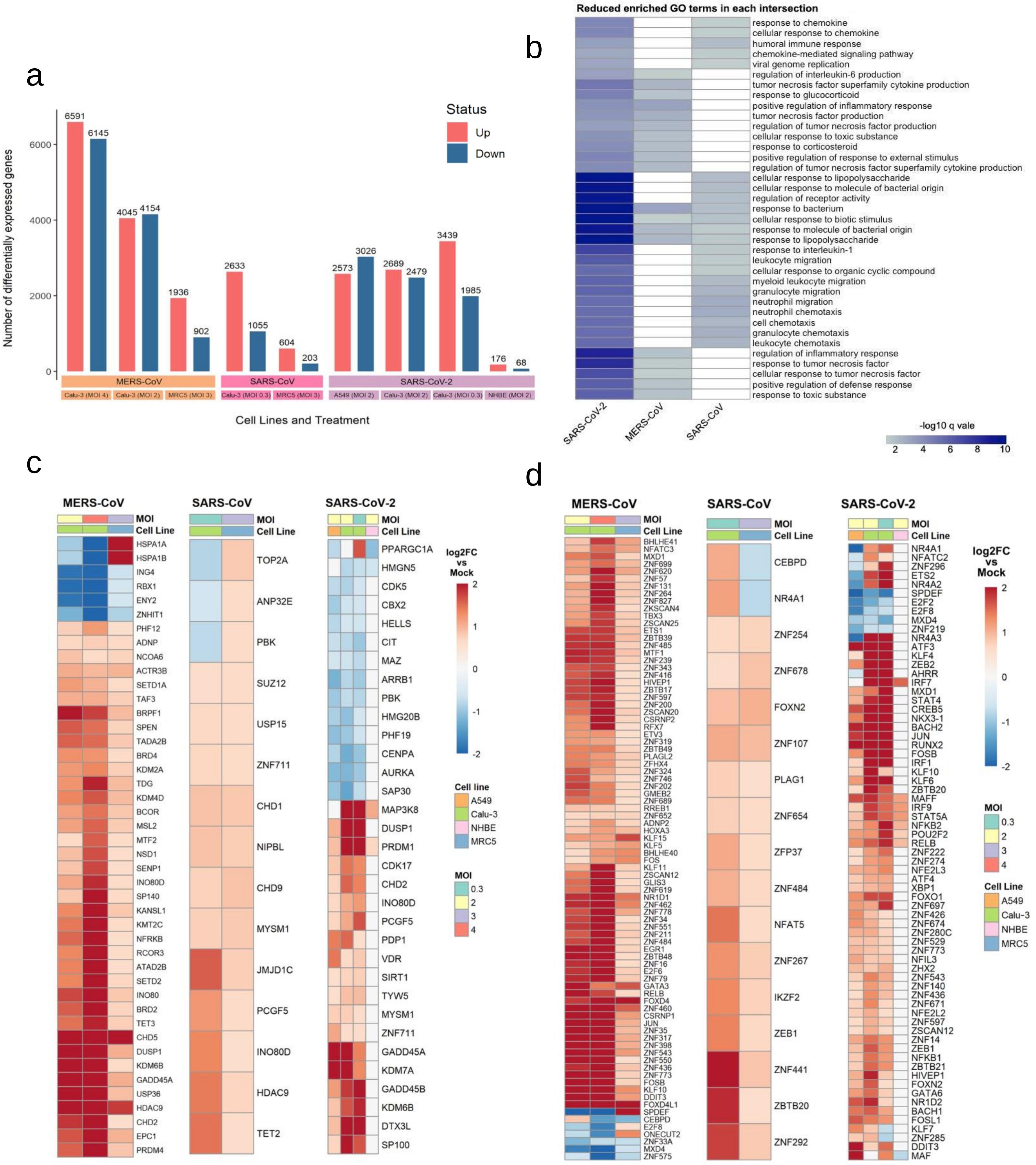

Supplementary Figure 1. Differential expression analysis of Coronaviruses infected cell lines.
a Number of up and down-regulated differentially expressed genes (DEGs) in the analyzed cell lines for each viral infection. **b** Reduced enriched GO terms shared between two or more virus-associated gene sets. **c** Log2 fold-change expression of SARS-CoV-2 virus-associated epifactors across different viral infections; blank color represents non-significant differential expression. **d** Log2 fold-change expression of SARS-CoV-2 virus-associated TFs across different viral infections; blank color represents non-significant differential expression.

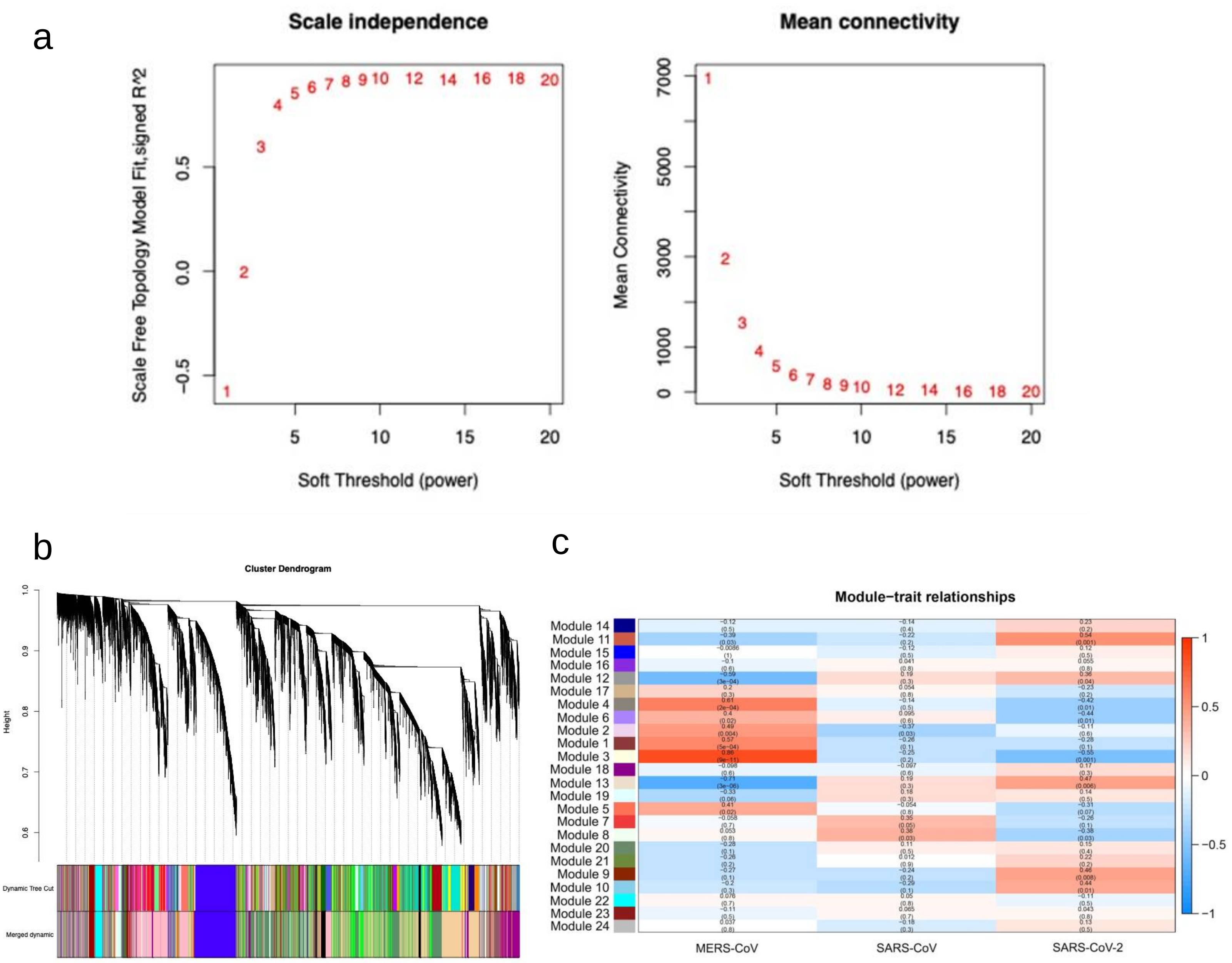

Supplementary Figure 2. Weighted Gene Co-expression Network. a Analysis of network topology for different soft-thresholding powers. **b** Clustering dendrogram of genes with their corresponding module colors before (Dynamic Cut Tree) and after (Merged Dynamic) similarity merging. **c** Module-virus association; the numbers inside each cell correspond to the correlation coefficient (top), which also dictates the cell color, and p value (bottom).

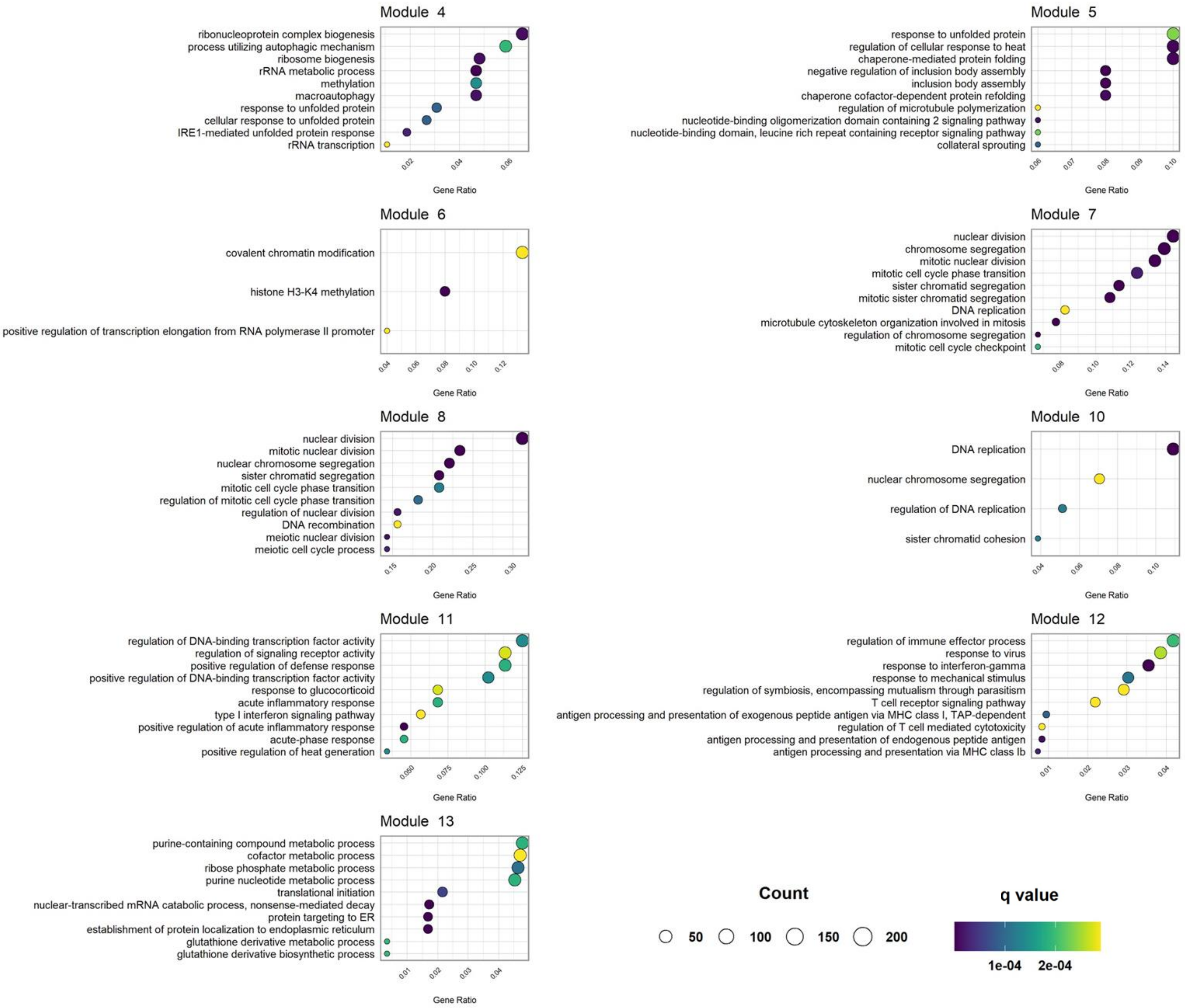

Supplementary Figure 3. Top 10 simplified enriched Gene Ontology terms of biological process in each of the relevant modules for coronaviruses infection. Go terms are ordered by q-value. Modules 1, 2, 3 and 9 are missing because they were not significantly enriched with any GO term.

a

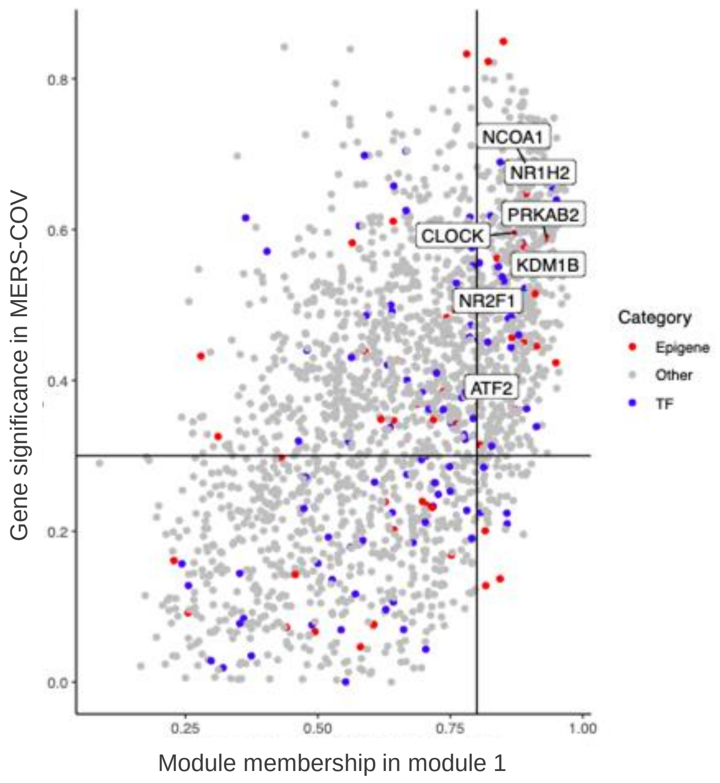

b

Gene	Function	Drug
NCOA1	Nuclear receptor coactivator / H3 and H4 HAT	Etoposide, Tamoxifen
NR1H2	Nuclear receptor / TF	Bexarotene, Diacerein, Alitretinoin, Isopropyl alcohol
PRKAB2	Regulatory subunit of AMPK	Metformin, Fostamatinib Acetylsalicylic acid
CLOCK	Regulation of circadian rhythms (TF)	Salbutamol
KDM1B	Histone lysine demethylase	Tranylcypromine
ATF2	TF / H2B and H4 HAT	Pseudoephedrine

c

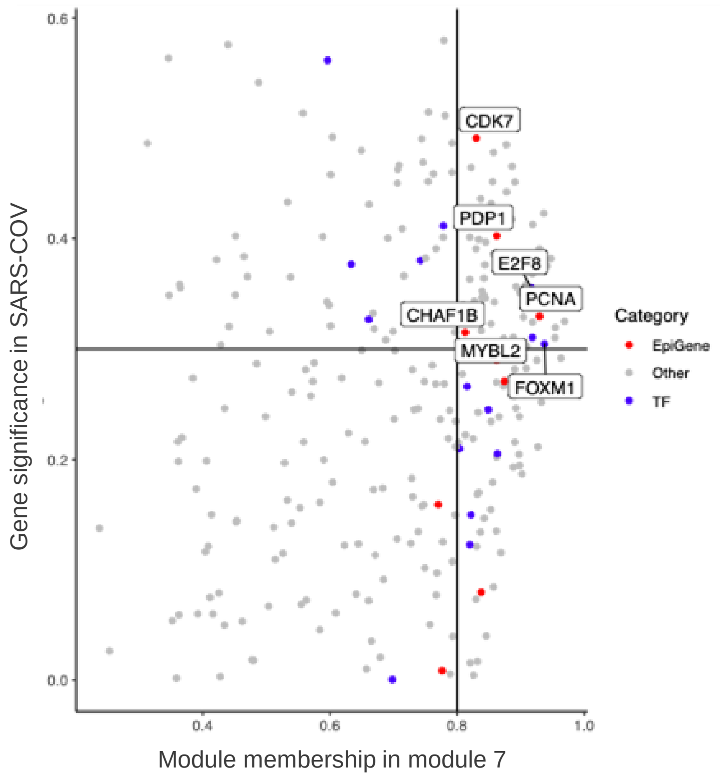

d

Gene	Function	Drug
CDK7	Regulator of cell cycle progression / TF	Nintedanib, Erlotinib, Dasatinib, Crizotinib, Sorafenib, Ruxolitinib, Tofacitinib, Palbociclib, Masitinib, Midostaurin, Sunitinib, Neratinib, Imatinib, Lapatinib, Afatinib, Pazopanib, Bosutinib, Axitinib, Vandetanib, Gefitinib, Nilotinib
PCNA	Cofactor of DNA polymerase delta	Liothyronine Acetylsalicylic acid

Supplementary Figure 4. Potential epigenetic therapeutic targets for MERS and SARS-CoV. a Module membership and Gene Significance of gene members of module 1. **b** Approved drugs for module 1's important epifactors and TFs. **c** Module membership and Gene Significance of gene members of module 7. d Approved drugs for module 7's important epifactors and TFs
